# A quality improvement initiative to improve operating room well-being: the Microaffirmations in Perioperative Personnel Project (The MAPP Project)

**DOI:** 10.1016/j.bjao.2026.100530

**Published:** 2026-02-03

**Authors:** Shanique B. Kilgallon, Vanessa Olbrecht, Abigail Storm, Loren Berman, William J. Parkes, Jacqueline L. Crawford, Holly Antal

**Affiliations:** 1 Department of Anesthesia, Nemours Children’s Health, Wilmington, DE, USA; 2 Departments of Anesthesia and Pediatrics, Sidney Kimmel Medical College of Thomas Jefferson University, Philadelphia, PA, USA; 3 Nemours Children’s Health Biomedical Research Informatics Center, Wilmington, DE, USA; 4 Department of Surgery, Nemours Children’s Health, Wilmington, DE, USA; 5 Division of Otolaryngology, Department of Surgery, Nemours Children’s Health, Wilmington, DE, USA; 6 Department of Perioperative Services, Nemours Children’s Health, Wilmington, DE, USA; 7 Nemours Children's Health, Jacksonville, FL, USA

**Keywords:** burnout, microaffirmations, microaggressions, operating room culture, perioperative team dynamics, professional fulfilment

## Abstract

**Background:**

Burnout and lack of professional fulfilment are prevalent among perioperative personnel, often exacerbated by a culture that lacks positive reinforcement. Microaffirmations—small, intentional acts of recognition—may offer a scalable strategy to improve workplace culture and well-being. The primary objective was to evaluate the impact of a microaffirmation-based intervention, the Microaffirmations in Perioperative Personnel Project (MAPP Project), on professional fulfilment and burnout. The secondary objective was to evaluate changes in perceived and experienced microaggressions among perioperative staff.

**Methods:**

A toolkit of microaffirmation examples was disseminated to perioperative members, and volunteer peer MAPP coaches modelled affirming behaviours. Surveys were administered at baseline before start of the intervention and at 3 and 6 months after the end of the intervention. The primary outcomes of professional fulfilment and burnout were measured using the validated Professional Fulfillment Index. Secondary outcomes including perceived and experienced microaggressions were assessed via a custom Microaggressions Impact Questionnaire.

**Results:**

A total of 388 responses were collected across the three time points. Mean fulfilment scores increased from 2.53 at baseline to 2.67 at 6 months (*P*=0.222), whereas burnout scores decreased from 1.06 to 0.87 (*P*=0.029). Perceived microaggressions and their reported impact also declined. Among 67 participants who completed all three surveys, trends were consistent but not statistically significant. Survey response rates were 63%, 40%, and 41% at each time point, respectively.

**Conclusions:**

The MAPP Project was associated with improved fulfilment, reduced burnout, and decreased perceived microaggressions among perioperative staff. These findings suggest that microaffirmation-based interventions may be a feasible and effective strategy to enhance workplace culture in health care settings.

Operating room culture is often shaped by institutional norms and unspoken rules, where silence is frequently interpreted as a sign of adequacy or success, reflecting a broader ‘no news is good news’ mentality that can obscure opportunities for feedback and improvement.^1^ This mindset frequently extends to interpersonal feedback, where positive reinforcement is rare and silence is assumed to indicate competence. The absence of consistent, affirming communication has been associated with increased professional dissatisfaction and burnout among perioperative personnel.^2^

To address this gap, our quality improvement (QI) project implemented the use of microaffirmations. First introduced by Rowe^3^ in 2008, microaffirmations have since gained traction in academic literature as a tool for fostering inclusion and psychological safety. They are defined as ‘apparently small acts, which are often ephemeral and hard-to-see, events that are public and private, often unconscious but very effective, which occur wherever people wish to help others to succeed’.^3^

The conceptual foundation for this intervention, including select examples from the microaffirmations toolkit, including three of the five edited toolkit examples used in the MAPP Project, has been described in a related published manuscript.^4^ That article references the existence of unpublished data from the MAPP Project, whereas this current manuscript presents the detailed analysis of survey data collected at multiple time points to evaluate the potential impact of the use of microaffirmations on professional fulfilment, burnout, and perceived microaggressions.

## Problem description

Perioperative environments are high-stakes, fast-paced settings where effective teamwork and psychological safety are essential for optimal patient care. However, interpersonal dynamics in these settings can be strained, particularly in diverse teams where subtle behaviours may impact inclusion and morale.

### Available knowledge

Microaffirmations are small, intentional acts that affirm and validate others that have been shown to foster inclusivity, enhance team cohesion, and improve workplace culture.^3^^,^^5^ Although their use has been explored in educational and corporate settings, their application in perioperative health care teams remains underreported.

### Rationale

Recognising the need for a more inclusive and affirming culture, a multidisciplinary team at a freestanding paediatric hospital initiated the Microaffirmations in Perioperative Personnel (MAPP) Project. The initiative aimed to leverage microaffirmations as a low-cost, scalable strategy to promote psychological safety and collegiality among perioperative staff.

### Specific aims

This QI project sought to implement and evaluate a three-pronged intervention: (1) the dissemination of a toolkit with occupationally relevant microaffirmation examples, (2) the engagement of perioperative leaders as MAPP coaches to model and promote affirming behaviours, and (3) reinforcement of efforts through the public recognition of affirmations via visual displays.

Ultimately, this project aimed to improve professional fulfilment, reduce burnout (primary outcomes), and mitigate the impact of microaggressions (secondary outcome) by embedding microaffirmation practices into the daily routines of perioperative staff. Through education, modelling, and visible reinforcement, the MAPP Project sought to enhance well-being and foster a more supportive operating room culture. We aimed to improve professional fulfilment, reduce burnout, and decrease perceived impact of both experienced and witnessed microaggressions through the intentional use of microaffirmations across the various silos of the perioperative setting.

## Methods

### Context

This QI initiative was conducted at a freestanding children’s hospital in the USA. The project received institutional QI approval and was deemed exempt from institutional review board (IRB) review. Institutional Quality Review Committee (QRC) approval was obtained, and explicit permission was received to collect Professional Fulfillment Index (PFI), Microaggressions Impact Questionnaire (MIQ), and participant data that were in the QRC application. Although this project was IRB-exempt, survey design and administration were reviewed for transparency and alignment with CHERRIES and CROSS reporting guidelines. The MAPP team comprised volunteer coaches from anaesthesiologists, surgeons, advanced practice nurses (APNs), certified registered nurse anaesthetists (CRNAs), surgical technicians, anaesthesiology technicians, and nurses. These coaches represented the perioperative groups from which survey responses would be collected.

The number and specialty distribution of active MAPP coaches evolved across survey periods. (1) Baseline (August 2024): six coaches—two surgeons, two anaesthesiologists, one nurse, and one anaesthesia technician. (2) Three months post-intervention (November 2024): 12 coaches—four surgeons, three anaesthesiologists, three nurses, and two anaesthesia technicians. (3) Six months post-intervention (February 2025): 15 coaches—all from 3 months post-intervention, in addition to one CRNA, one surgical technician, and one APN.

This expansion reflected increased institutional engagement and broader representation across perioperative roles.

This manuscript was prepared using the SQUIRE 2.0 reporting guidelines for QI studies.

### Intervention

The MAPP Project aimed to foster a more affirming and inclusive perioperative culture through a three-part intervention.

(1) Microaffirmations toolkit. A microaffirmations toolkit (Supplementary material) was developed to illustrate three key behaviours: (i) give people credit by acknowledging their contributions, (ii) offer support and stand up for people when they are being discredited or demeaned, and (iii) provide positive feedback that helps everyone recognise and build on their strengths. The toolkit included five example microaffirmations and was disseminated via one-page flyers posted throughout non-sterile perioperative areas. Distribution was further supported by the hospital’s Employee Well-Being Wagon, staffed by volunteer MAPP coaches and well-being personnel, during all survey periods. This mobile engagement strategy provided printed toolkits, QR codes for digital access, and opportunities for in-person interaction with MAPP team members to reinforce the intervention’s visibility and accessibility.
(2) Leadership engagement. Perioperative leaders were enlisted as MAPP coaches to model and promote the use of microaffirmations within their teams. Coaches introduced the initiative through brief presentations during rounds or meetings, shared toolkit examples, and encouraged survey participation. The full MAPP team met regularly (one to two times per month) to review feedback and adapt implementation strategies. For example, based on early feedback, additional flyers were created to publicly recognise team members who demonstrated affirming behaviours.
(3) Public ‘Microaffirmations in Action’ flyers. To promote a culture of psychological safety and recognition within the perioperative environment, we implemented a visual intervention titled ‘Microaffirmations in Action’, flyers that were distributed on a bimonthly to monthly basis and displayed in non-sterile perioperative areas. Each flyer featured staff members who had either given, received, or witnessed a microaffirmation. With previous consent, photographs and names of individuals were included to personalise and amplify the impact of the recognition. To facilitate ongoing participation, a QR code linking to the nomination form was prominently displayed on separate flyers in similar non-sterile locations. This approach aimed to normalise affirming behaviours and encourage peer-to-peer acknowledgment in high-stress clinical settings.

### Study of the intervention

Surveys were distributed electronically to all perioperative personnel at three time points: baseline (pre-intervention), 3 months post-intervention, and 6 months post-intervention. Each survey remained open for 2 weeks. Data were collected and managed using REDCap, a secure, web-based platform designed for validated data capture and audit tracking.^6^^,^^7^ The survey process and intervention refinement were guided by the Plan-Do-Study-Act (PDSA) framework, which was applied iteratively throughout the project to support continuous learning and adaptation based on feedback and observed outcomes. The MAPP interventions described above were continued throughout the administration of both post-intervention surveys, and at this time the plan is to continue them indefinitely.

### Measures

Primary outcome measures were professional fulfilment and burnout, assessed using the validated 16-item PFI.^8^ The PFI includes three subscales: professional fulfilment (six items), interpersonal disengagement (six items), and work exhaustion (four items). Each item is scored from 0 to 4 based on the associated 5-point Likert scale. Professional fulfilment is calculated by taking the averages of the six items within the professional fulfilment subscale, and burnout is then calculated by taking the average of the six items that make up interpersonal disengagement and the four items that make up work exhaustion. Secondary outcome measures included perceived microaggressions, assessed via a novel MIQ composed of four items rated on a 4-point Likert scale (never to frequently), with an option to respond ‘prefer not to say’. A standardised definition for microaggression and microaffirmation was presented at the beginning of the MIQ survey to provide a grounding context for all participants. The MIQ was developed by the first author to assess the perceived impact of microaggressions in the perioperative setting. It was designed to capture both personal and observed experiences. The MIQ Likert scale items were ranked 1–4, and the sum of the values was used for analysis in addition to looking at the 1–4 rank of each item. Although not formally validated before implementation, *post hoc* analyses of internal consistency and construct validity are underway. Participant data collected included occupation, role, race, ethnicity, gender, and years of experience. Participation in all surveys was voluntary, and responses were collected anonymously to ensure confidentiality.

### Analysis

Analyses were conducted on two cohorts: (1) all survey responses at each time point and (2) responses from individuals who completed all three surveys. Fulfilment and burnout scores were calculated using established thresholds (≥3.0 for fulfilment, ≥1.33 for burnout). The MIQ scores were aggregated across the four items.

### Statistical analysis

Descriptive statistics were used to assess distribution (skewness and kurtosis). Categorical variables (e.g. role, race, gender) were analysed using χ^2^ tests. Paired-sample *t*-tests compared outcomes across time points. Repeated-measures analysis of variance (anova) assessed changes over time. Additional analyses included anova for years of experience and Kruskal–Wallis tests for role, race, ethnicity, and gender. Pearson’s correlation was used to examine relationships among fulfilment, burnout, and MIQ scores. A *P*-value <0.05 was considered statistically significant. All analyses were conducted using SPSS Statistics, version 27 (IBM Corp., Armonk, NY, USA).

## Results

### Survey responses

An e-mail invitation for the REDCap survey was sent to 267 participants at each time point with 169 responses (63%) at baseline, 109 responses (40%) at 3 months post-intervention, and 110 responses (41%) at 6 months post-intervention. At the conclusion of the 6-month intervention, 68% (*n*=177) of the participant population completed at least one survey across the three time points. A detailed breakdown of patient characteristics can be found in Table 1. Some respondents did not complete the PFI (baseline, *n*=7; 3 months post-intervention, *n*=3; and 6 months post-intervention, *n*=4). Some respondents also did not complete all of the MIQ questions (baseline, *n*=12; 3 months post-intervention, *n*= 6; and 6 months post-intervention, *n*=8). This difference in response rate for PFI and MIQ resulted in small variations in sample size across analyses.

**Table 1:** Frequencies of patient characteristics for the entire survey by time points. APN, advanced practice nurse; CRNA, certified registered nurse anaesthetist.

	Baseline	3 Months post-intervention	6 Months post-intervention
	*n* (%)	*n* (%)	*n* (%)
Role			
Anaesthesia tech	6 (3.6)	3 (2.8)	2 (1.8)
Anaesthesiologist	23 (13.6)	15 (13.8)	10 (9.1)
APN	10 (5.9)	9 (8.3)	16 (14.5)
CRNA	16 (9.5)	9 (8.3)	12 (10.9)
Nurse	64 (37.9)	42 (38.5)	32 (29.1)
Surgeon	34 (20.1)	25 (22.9)	30 (27.3)
Surgical tech	10 (5.9)	4 (3.7)	5 (4.5)
Other	4 (2.4)	2 (1.8)	3 (2.7)
Prefer not to say	2 (1.2)	0 (0.0)	0 (0.0)
Race			
American Indian	1 (0.6)	1 (0.9)	0 (0.0)
Asian	14 (8.3)	9 (8.3)	11 (10.0)
Black	16 (9.5)	7 (6.4)	6 (5.5)
White	121 (71.6)	83 (76.1)	82 (74.5)
Prefer not to say	17 (10.1)	9 (8.3)	11 (10.0)
Ethnicity			
Hispanic/Latino/Spanish origin	8 (4.7)	2 (1.8)	4 (3.6)
Not Hispanic/Latino/Spanish origin	141 (83.4)	93 (85.3)	92 (83.6)
Prefer not to say	13 (7.7)	9 (8.3)	10 (9.1)
Missing	7 (4.1)	5 (4.6)	4 (3.6)
Gender			
Female	130 (76.9)	82 (75.2)	78 (70.9)
Male	34 (20.1)	21 (19.3)	25 (22.7)
Other	0 (0.0)	1 (0.9)	0 (0.0)
Prefer not to say	5 (3.0)	4 (3.7)	7 (6.4)
Missing	0 (0.0)	1 (0.9)	0 (0.0)
Years in practice			
0–5 yr	33 (19.5)	22 (20.2)	15 (13.6)
6–10 yr	20 (11.8)	10 (9.2)	12 (10.9)
11–15 yr	34 (20.1)	24 (22.0)	28 (25.5)
16+ yr	79 (46.7)	53 (48.6)	52 (47.3)
Prefer to not say	3 (1.8)	0 (0.0)	3 (2.7)

### Statistical analysis

Mean fulfilment score increased across all three time points: 2.53 (sd, 0.67) at baseline, 2.64 (sd, 0.72) at 3 months post-intervention, and 2.67 (sd, 0.75) at 6 months post-intervention (*P*=0.222) (see Table 2). Mean burnout score decreased across baseline through 6 months post-intervention: 1.06 (sd, 0.62), 0.92 (sd, 0.59), and 0.87 (sd, 0.57), respectively. MIQ question 1 (*P*=0.088), ‘I personally experience microaggressions in the perioperative space’, question 2 (*P*=0.125), ‘I witness others being affected by microaggressions in the perioperative space’, question 3 (*P*=0.654), ‘Experienced microaggressions negatively impact my daily work’, and question 4 (*P*=0.587), ‘Witnessed microaggressions negatively impact my daily work’, all saw a decrease from the initial baseline value. There was a significant average difference among burnout across the time points (*P*=0.029).

**Table 2:** Mean and standard deviation of outcome measures of interest. Anova, analysis of variance. ^∗^Fulfilment reference range >3.00. ^†^Burnout reference range ≥1.33.

		*n*	*μ*	*σx*	anova *P*-value
Fulfilment^∗^	Baseline	162	2.53	0.67	0.222
	3 months post-intervention	106	2.64	0.71	
	6 months post-intervention	106	2.67	0.75	
Burnout^†^	Baseline	162	1.06	0.62	0.029
	3 months post-intervention	106	0.92	0.59	
	6 months post-intervention	106	0.87	0.57	
I personally experience microaggressions in the perioperative space.	Baseline	157	2.56	0.96	0.088
	3 months post-intervention	103	2.35	0.86	
	6 months post-intervention	102	2.35	0.80	
I witness others being affected by microaggressions in the perioperative space.	Baseline	157	2.8	0.92	0.125
	3 months post-intervention	103	2.78	0.79	
	6 months post-intervention	102	2.59	0.76	
Experienced microaggressions negatively impact my daily work.	Baseline	157	2.36	0.98	0.654
	3 months post-intervention	103	2.27	0.88	
	6 months post-intervention	102	2.27	0.87	
Witnessed microaggressions negatively impact my daily work.	Baseline	157	2.44	0.96	0.587
	3 months post-intervention	103	2.38	0.85	
	6 months post-intervention	102	2.32	0.82	

### Cohort survey responses

Data from the 67 (25.1%) individuals who completed the survey at baseline, 3 months post-intervention, and 6 months post-intervention were analysed as a separate cohort. The separation of this cohort was intended to assess changes across the time points. Surgeons and nurses made up a majority of this cohort with 24 (35.8%) and 21 (31.3%), respectively. More than three-quarters of the cohort identified as White (*n*=52), 88.1% identified as not Hispanic/Latino/Spanish origin (*n*=59), 21 (31.3%) identified as male, 46 (68.7%) as female, with none identifying as other gender (Table 3). Some respondents did not complete all the MIQ questions (baseline, *n*=1; 3 months post-intervention, *n*=2; and 6 months post-intervention, *n*=2), resulting in small variations in sample size across analyses. There was a statistically significant difference between gender (male/female) and MIQ responses across the three time points (*P*=0.025). Those who identified as female had a statistically significant difference in ‘I personally experience microaggressions in the perioperative space’ (*P*=0.003), ‘I witness others being affected by microaggressions in the perioperative space’ (*P*<0.001), and ‘Witnessed microaggressions negatively impact my daily work’ (*P*=0.005) at baseline, 3 months post-intervention, and 6 months post-intervention. Those who identified as male had statistically significant difference in ‘I witness others being affected by microaggressions in the perioperative space’ (*P*<0.001) at baseline, 3 months post-intervention, and 6 months post-intervention. Specific frequency breakdown of the cohort’s responses to the MIQ questions can be found in Table 4.

**Table 3:** Categorical breakdown of the characteristics of the cohort who completed all three time points. NP, nurse practitioner; PA, physician assistant. ∗*P*<0.05 between gender and Microaggressions Impact Questionnaire (categorically).

	*n* (%)
Role	
Anaesthesiologist	10 (14.9)
Surgeon	24 (35.8)
Advanced practice provider (NP/PA)	6 (9.0)
Certified registered nurse anaesthetist	4 (6.0)
Nurse	21 (31.3)
Surgical technician	2 (3.0)
Race	
Asian	9 (13.4)
Black or African American	3 (4.5)
White	52 (77.6)
Prefer not to answer	3 (4.5)
Ethnicity	
Hispanic/Latino/Spanish origin	4 (6.0)
Not Hispanic/Latino/Spanish origin	59 (88.1)
Prefer not to answer	2 3.0)
Missing	2 (3.0)
Gender∗	
Female	46 (68.7)
Male	21 (31.3)
Practice	
0–5 yr	7 (10.4)
6–10 yr	11 (16.4)
11–15 yr	13 (19.4)
16+ yr	36 (53.7)

**Table 4:** Descriptives and frequencies for outcomes of interest (burnout, fulfilment, MIQ) in the cohort who completed all three time points. MIQ, Microaggressions Impact Questionnaire. ^∗^Reference threshold for fulfilment ≥3.00. ^†^Reference threshold for burnout ≥1.33. ^‡^*P*-value in repeated-measures analysis of variance.

	Baseline		3 Months post-intervention		6 Months post-intervention		
	$\bar{X}$	$\sigma_{x}$	$\bar{X}$	$\sigma_{x}$	$\bar{X}$	$\sigma_{x}$	*P*-value ^‡^
Fulfilment^∗^	2.62	0.69	2.71	0.75	2.73	0.82	0.155
Burnout^†^	1.04	0.60	0.88	0.60	0.84	0.61	0.336
MIQ	9.47	3.20	9.11	2.85	8.95	2.79	0.078

	***n***	**%**	***n***	**%**	***n***	**%**	

Personally experienced microaggressions							0.342
Never	12	17.90	14	20.90	13	19.40	
Rarely	26	38.80	31	46.30	33	49.30	
Occasionally	20	29.90	16	23.90	16	23.90	
Frequently	9	13.40	4	6.00	3	4.50	
Missing	0	0.00	2	3.00	2	3.00	
Saw others experience microaggressions							0.134
Never	6	9.00	3	4.50	6	9.00	
Rarely	20	29.90	24	35.80	27	40.30	
Occasionally	29	43.30	31	46.30	26	38.80	
Frequently	11	16.40	7	10.40	6	9.00	
Prefer not to answer/missing	1	1.50	2	3.00	2	3.00	
Experience of microaggressions negatively impacted work							0.228
Never	18	26.90	17	25.40	16	23.90	
Rarely	25	37.30	30	44.80	29	43.30	
Occasionally	18	26.90	14	20.90	17	25.40	
Frequently	5	7.50	4	6.00	3	4.50	
Prefer not to answer/missing	1	1.50	2	3.00	2	3.00	
Witnessing others experience of microaggressions negatively impacted work							0.446
Never	16	23.90	13	19.40	12	17.90	
Rarely	22	32.80	27	40.30	28	41.80	
Occasionally	24	35.80	22	32.80	24	35.80	
Frequently	4	6.00	3	4.50	1	1.50	
Prefer not to answer/missing	1	1.50	2	3.00	2	3.00	

Among women, fulfilment at baseline had a strong positive and significant correlation with fulfilment at 3 months post-intervention (*r*=0.683, *P*<0.01) and 6 months post-intervention (*r*=0.712, *P*<0.01); a strong negative significant correlation with burnout at baseline (*r*=−0.551, *P*<0.01), 3 months post-intervention (*r*=−0.559, *P*<0.01), and 6 months post-intervention (*r*=−0.471, *P*<0.01); and a moderately strong negative significant correlation with overall MIQ combined score at baseline (*r*=−0.376, *P*<0.05), 3 months post-intervention (*r*=−0.417, *P*<0.01), and 6 months post-intervention (*r*=−0.382, *P*<0.05). Compared with men, fulfilment at baseline had a significantly moderate positive correlation with fulfilment at 3 months post-intervention (*r*=0.444, *P*<0.05), a significantly strong positive correlation with fulfilment at 6 months post-intervention (*r*=0.734, *P*<0.01), and a significantly strong negative correlation with burnout at baseline (*r*=−0.529, *P*<0.05). Men also had a significantly strong positive correlation with MIQ overall score at 3 months post-intervention with burnout at baseline (*r*=0.528, *P*<0.05), 3 months post-intervention (*r*=0.0646, *P*<0.01), and 6 months post-intervention (*r*=0.638, *P*<0.01). Fulfilment and burnout across baseline, 3 months post-intervention, and 6 months post-intervention appeared to have stronger negative correlations among women, and burnout and MIQ total score across the time points had a strong positive correlation among men. Subgroup analysis of baseline burnout scores revealed that anaesthesiologists and surgical technicians had burnout scores at the burnout threshold (≥1.33), suggesting elevated risk in these roles.

**Fig 1:**
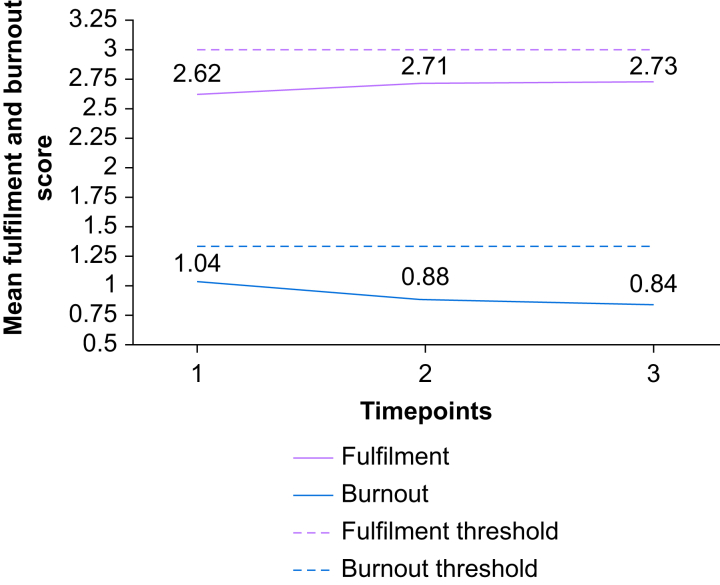
Mean fulfilment and burnout of study population by timepoint.

Intervention impact of the QI project on this perioperative personnel cohort is summarised and outlined in detail in Table 2 (see Fig. 1 for visual summary). There was a statistically significant difference in burnout (decreasing) from baseline to 6-month follow-up in the entire survey population (*P*=0.029). Although the responses on MIQ question 1, ‘I personally experience microaggressions in the perioperative space’, were not statistically significant from baseline to 6-month follow-up (*P*=0.088), the reduction in perceived personal microaggression highlights the impact of the QI project.

For participants who completed all three surveys, there was a non-significant trending decrease in burnout, improvement of fulfilment scores, and a decrease in the impact of microaggressions.

## Discussion

This QI initiative demonstrated that the implementation of a microaffirmations toolkit, supported by visible reinforcement strategies such as public recognition flyers, was associated with improvements in professional fulfilment and reductions in both burnout and the perceived impact of microaggressions among perioperative personnel. These improvements were observed as early as 3 months after implementation and continued through the 6-month follow-up. However, subgroup analysis revealed that anaesthesiologists and surgical technicians were at or near the burnout threshold at baseline underscoring the importance of targeted well-being strategies for specific roles. This finding may offer a proactive approach to mitigating potential retention risks.

Survey response rates were 63%, 40%, and 41% at baseline, 3 months, and 6 months, respectively—substantially higher than the commonly cited 25% threshold for acceptable response rates in health care surveys.^9^, ^10^, ^11^ This suggests strong institutional engagement and interest in the intervention.

One likely contributor to the success of this initiative was the deliberate engagement of perioperative leadership across specialties before project launch. Department chairs and executive leaders were consulted and asked to nominate respected individuals within their teams to serve as MAPP coaches. These coaches played a critical role in disseminating the toolkit, modelling microaffirmation behaviours, and maintaining consistent messaging across teams. Additionally, the hospital’s Center for Associate Well-Being supported the initiative by deploying a ‘well-being wagon’ during survey periods, which provided printed toolkits, QR codes for digital access, and in-person engagement with MAPP team members. This multifaceted approach ensured accessibility across shifts and reinforced the project’s visibility and credibility.

The MAPP Project was designed to be low cost and minimally disruptive to clinical workflows. All activities were conducted using discretionary time volunteered by MAPP coaches. Coaches introduced the initiative during existing team huddles and meetings, typically requiring 5–10 min per session. To support consistency, the first author created optional PowerPoint slides for coaches to use during these brief engagements. The MAPP team met one to two times per month to discuss microaffirmation concepts, current opportunities related to the project, and anticipated questions from perioperative staff. Participation in meetings and reinforcement activities such as staffing the well-being wagon was entirely voluntary. Coaches who chose to assist with the well-being wagon typically signed up for 20- to 30-min blocks that fit their clinical schedules. For those unable to attend meetings, the first author provided individualised updates to ensure continuity.

Although improvements in burnout scores were statistically significant across the full sample, subgroup analyses limited to participants who completed all three survey waves did not reach statistical significance. This may be attributable to reduced statistical power from smaller sample sizes or variability in individual experiences over time. Nevertheless, the consistent positive trend across time points suggests that improvements were likely occurring at the individual level and were not solely attributable to changes in the respondent pool. Similarly, although professional fulfilment scores did not show statistically significant improvement over time, the upward trend observed across the full cohort and among most specialty groups reflects the overall positive impact of the intervention. Notably, although microaffirmations have previously been described as a strategy to mitigate microaggressions, this study is among the first to explore their potential influence on professional fulfilment and burnout within a perioperative clinical setting.

These findings align with the broader literature in positive psychology, which suggests that frequent experiences of positive affect can foster resilience, goal-directed behaviour, and well-being.^12^ Microaffirmations—brief, intentional acts of recognition—may serve as a form of prosocial motivation, which has been shown to enhance employee well-being, job performance, and career satisfaction.^13^ Moreover, positive psychology interventions in organisational settings have demonstrated promise in reducing burnout and stress while promoting engagement and fulfilment.^14^

Although the association between microaffirmations and improvements in burnout and professional fulfilment is relatively novel, it is reasonable to hypothesise an indirect relationship between microaffirmations and broader organisational outcomes. Burnout is well established as a contributor to increased turnover and disengagement among health care professionals. Among surgeons, burnout has been linked to greater intent to leave clinical practice and diminished career satisfaction.^15^ Afonso and colleagues^16^ demonstrate an increased likelihood of leaving practice among anaesthesiologists who were at risk of burnout or who suffered from burnout syndrome. Similar associations have been observed among nurse anaesthetists, where burnout correlates with increased intent to leave the profession.^17^ Nurses working in high-stress environments also report higher turnover and lower engagement when experiencing burnout.^18^ The financial implications of turnover are substantial; the average cost to onboard a perioperative clinician varies by subspecialty but may reach several hundred thousand dollars per provider.^19^

Unlike many clinician well-being interventions that require substantial financial investment, this QI initiative was implemented at minimal cost. Although the exact financial outlay was not quantified, expenses were limited to printing and posting flyers. More critically, the success of the intervention hinged on voluntary participation from individuals committed to fostering a more supportive professional environment. This effort extended beyond general collegiality by instead emphasising intentional and affirming actions across perioperative subspecialties. Although this represents discretionary effort, participants expressed a willingness to continue their involvement, as evidenced by the tangible and positive measurable impact observed at the pilot site—an impact substantiated by data-driven outcomes.

### Limitations

This QI project has several limitations. First, although the overall survey response rates were high for health care-based initiatives, the proportion of participants who completed all three time points was smaller, potentially limiting the power of longitudinal comparisons. Second, participation in the intervention and survey was voluntary, introducing the possibility of selection bias; individuals more engaged or positively inclined toward the initiative may have been more likely to participate and report favourable outcomes. Third, the study was conducted at a single paediatric institution, which may limit generalisability to other health care settings with different organisational cultures or staff compositions. Fourth, although the PFI is a validated tool, the MIQ was developed specifically for this QI project to capture relevant perceptions of microaggressions and has not been formally validated. As such, findings related to microaggressions should be interpreted with caution. Finally, although the intervention was associated with improvements in professional fulfilment and reductions in burnout and perceived microaggressions, causality cannot be established because of the absence of a control group. Future studies using randomised or controlled designs across multiple institutions are warranted to further evaluate the effectiveness and scalability of microaffirmation-based interventions, although the feasibility of such a study is quite low.

### Potential future directions

Future directions for the MAPP Project include expanding the scope of evaluation tools to better capture team dynamics and organisational-level influences on well-being. The Well-Being Influencers Survey for Healthcare (WISH) metric, developed by Higgins and colleagues,^20^ offers a validated framework for assessing workplace support and may complement the individual-level insights provided by the PFI and MIQ. Preliminary discussions are underway to explore its integration into future phases of the initiative, including the next iteration, ‘MAPP 2.0’.

### Conclusions

The MAPP Project represents a novel, grassroots approach to shifting operating room culture through the intentional use of microaffirmations. While the PDSA cycle continues, early findings suggest that solution-oriented strategies that engage the full spectrum of perioperative personnel can meaningfully influence workplace culture.

This initiative demonstrated that with strong engagement, leadership support, and a bottom–up implementation model, such interventions may be adaptable to other clinical and non-clinical environments. By fostering a culture of recognition and inclusion, microaffirmations may contribute to broader institutional goals, including enhanced employee satisfaction, improved retention, and, ultimately, patient safety.

Future work should explore the role of microaffirmations in addressing other dimensions of employee well-being, such as emotional exhaustion and workplace disengagement. Another area of opportunity is to investigate why fulfilment and burnout may have a stronger negative correlation among women, whereas burnout and MIQ score have a strong positive correlation among men regardless of occupational role. Although some outcomes are difficult to quantify, anecdotal feedback, such as a senior surgeon attributing their decision to remain at the institution to the hope inspired by this project, underscores the potential human impact of such efforts.

In moving away from the long-standing mantra of ‘no news is good news’, the MAPP Project helped cultivate a new narrative: ‘good news is good news’. This cultural shift, although difficult to quantify, may be among the most meaningful outcomes of the initiative.

## Authors’ Contributions

Designed and submitted the QI proposals, recruited MAPP coaches and team members, developed the REDCap questionnaire, curated relevant literature for team review, led manuscript development, toolkit example creation, originator of the MIQ: SBK

Supported initial REDCap design, executive leadership team liaison for project approval: VO

QI project design: VO, HA

Substantial manuscript editing: VO, HA

Co-developed the original toolkit example: VO, LB, HA

Helped shape the strategic direction of the intervention: VO, LB

Project design, REDCap cadence development: VO, LB, JLC, HA

Project statistician, responsible for all statistical analyses, development of figures and tables: AS

Served as the lead MAPP Coach for cross-specialty surgical team engagement and delivered multiple team presentations, led cross-specialty surgical team engagement through targeted presentations, coordinated team buy-in: LB

Manuscript development through structural editing: LB, WJP

Manuscript development through content refinement: LB, WJP, JLC

Served as a MAPP Coach: WJP, JLC

Played a key role in departmental engagement, particularly within otolaryngology, by delivering post-test presentations and facilitating team buy-in, manuscript development through content alignment with project goals: WJP

Led nursing team engagement—the largest perioperative subgroup—through targeted presentations and coordinated public recognition flyer placement in alignment with facility guidelines, contributed to toolkit development, assisted in compiling relevant literature, manuscript development through content organisation: JLC

Identified the PFI as the primary well-being metric, facilitated access to Delaware well-being resources, including the Well-Being Wagon: HA

## Data availability statement

Deidentified data that support the findings of this study are available from the corresponding author upon reasonable request.

## Declarations of interest

SBK is an Educational Consultant for Medtronic, Inc. The other authors declare that they have no conflicts of interest.
