# Supplementary material for: A quality improvement initiative to improve operating room well-being: the Microaffirmations in Perioperative Personnel Project (The MAPP Project)
Source: BJA Open. 2026 Feb 3;17:100530. doi: 10.1016/j.bjao.2026.100530 (PMC12936672; doi:10.1016/j.bjao.2026.100530)
Supplement: Multimedia component 1 [file mmc1.doc]

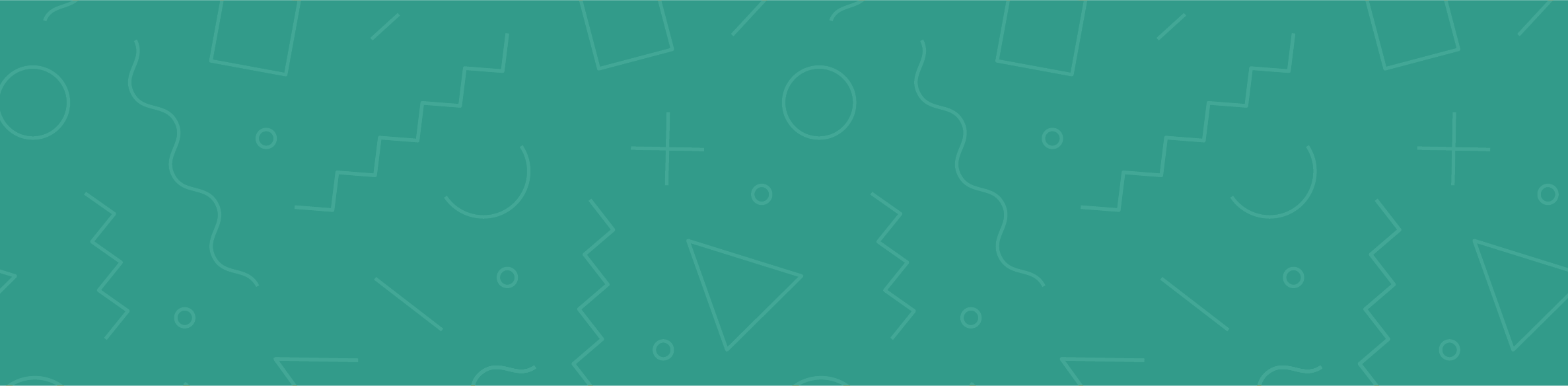


**MAPP Toolkit**

**Case 1: Illustrating Microaffirmation Principle of Giving People Credit by Acknowledging Contributions**

**a.** **Clinical Scenario**: A 16yoF is scheduled for Botox injection for a facial keloid. During the pre-op phone call, the severity of anxiety was discussed and there was a decision made to pre-assign members of her care team including the Anesthesiologist, Dr. A, and the Circulating RN, Bella. Typically, in preop holding, the only members of intraoperative care team that meet the patients are those from the surgical and anesthesiology team.

Based on the concern for severe anxiety, the circulating nurse, Bella, goes to Preop and introduces herself and strikes up a conversation with the patient prior to arrival to the operating room.

After witnessing this interaction, Dr. A, the Anesthesiologist says to Nurse Bella,

**Tool Kit Response Example:** " I really appreciated you going above and beyond to introduce yourself to this child ahead of time. Meeting the patient in preop was not in your usual scope of practice, but I am sure it will have a positive impact on her experience. So, thank you, because you would have already become an been an integral part of that positive experience.”

**b. Teaching point**: When Dr. A acknowledges Bella’s discretionary effort to help decrease this child’s anxiety, Bella’s role as an important team member in this child’s care is reinforced beyond her duties as a circulating RN.

**Case 2: Illustrating Microaffirmation Principle of Providing Positive Feedback that Helps Everyone Recognize and Build on their Strengths.**

**a.** **Clinical Scenario**: Dr. T is the sedation provider sedating a 6yo for a brain MRI when the patient starts to cough and has a precipitous decline in the oxygen levels. Help was called overhead. Four providers come in including the rotating ER Fellow, two Sedation Registered nurses and the Anesthesiologist. Dr. K, announces herself as the Anesthesiologist and offers up suggestions to Dr. T in the management of the child.

After the child is stabilized and case is rescheduled, Dr. T approaches Dr. K in the recovery area where the charge nurse and the Physician Division Chief of Sedation Services is present.

**Tool Kit Response Example**: Dr. T says, “Every time I am in the Sedation unit, and I see your name posted, I am immediately relieved by your presence. You consistently are calm, helpful, and supportive during every and any critical situation. Thank you for coming to help me handle the tenuous sedation case. I value your expertise.”

**b. Teaching point** By affirming Dr. K’s expertise in the presence of one of her division chiefs by her colleague of equal rank publicly reinforces her value to the clinical team to a supervisor who may not have the chance to consistently see her work on a day-to-day basis.

**Case 3: Illustrating Microaffirmation Principle of Offering Support and Standing Up for People If They Are Discredited or Demeaned.**

**a. Clinical Scenario**: A 4-month-old male is brought to the operating room for a pyloromyotomy given concerns for pyloric stenosis. After induction of anesthesia, the surgical time out is being completed by the Attending Surgeon, the team was encouraged to voice concerns if there are any. At this point, the Student Nurse Anesthetist, Jack, voices the concern that the patient’s temperature is 35.4 degrees Celsius and asks the circulating RN to raise temperature of the room given the concern for hypothermia.

The Surgical Attending, Dr. Z responds by saying, “It’s already too hot in here.”

**Tool Kit Response Example**: The circulating nurse, Bridget, interjects and says, “Jack is right, the patient is too cold Dr. Z, I will increase the room temperature, it's the right thing to do for the baby.”

**b. Teaching point:** Trainees can easily be intimidated to speak up given their trainee status, when Bridget reinforcing that Jack’s concerns were appropriate to keep the baby safe, she publicly encourages Jack to continue to be a patient advocate and encourage safe clinical practices, even if that means bringing up the concern to an experienced provider like a surgical attending.

**Case 4: Illustrating Microaffirmation Principle of Offering Support and Standing Up for People If They Are Discredited or Demeaned.**

**a. Clinical Scenario:** Several operating room staff members (nurses and techs) are in the OR lounge, after a long day of work, discussing how poorly they felt the day went because the OR charge nurse didn't assign them a morning or an afternoon break.  There is commentary about the charge nurse's perceived inability to run the board effectively. Nurse L says, "The OR charge nurse just does not know what she's doing"... "I know she plays favorites"...  "Can you believe she let Felicia go early?"

Nurse C overhears the staff's negative comments and conversations and is appalled at the comments being made.

**Tool Kit Response Example**: Nurse C says to the OR staff, “Show the OR charge nurse some grace.  There were several behind-the-scenes events that she navigated quite expertly including a level B trauma call, an employee with a needle stick and 5 callouts today.  Unfortunately, as Charge, not everyone can appreciate all the moving parts. She had a tough day, we should encourage her.”

**b. Teaching Point:**  Nurse C stood up for and was supportive of the OR charge nurse.  She immediately stopped the team from demeaning and discrediting the OR charge nurse. In standing up for the OR charge nurse, the staff's opinion of the OR charge nurse shifted for the good. They were provided a different perspective that they may not have had prior.  The integrity of the OR charge nurse remained intact, while the team's mindset about the OR charge nurse was placed on reset.

**Case 5: Illustrating Microaffirmation Principle of Provide Positive Feedback that helps Everyone Recognize and Build on Their Strengths**

**a. Clinical Scenario:** There is a difficult case involving lots of unanticipated equipment needs.  To complicate things, there is a scrub tech in training.  The team gets flustered but makes consistent dedicated effort. While the equipment is being assembled, the training scrub tech is often not assembling the instruments properly.  The surgeon gets impatient at times and eventually pauses the case and shows the learner how to do it right. After the case, the surgeon acknowledges the great hustle and appreciation for the team.

**Tool Kit Response Example:**

The surgeon says to the training tech, “this was a tough case to learn on, but you persisted, and it will become easier. Thank you for your tenacity during this case.”

While the surgeon is scrubbing out to the finished case, the supervising tech says to the surgeon, “Thank you for being patient and taking the time to teach during a tough moment.”

**b. Teaching Point:** The operating room is unpredictable, and things don’t always go as planned, this places additional burden on the team, and they may leave the case feeling like they didn’t perform well but a simple comment after the case when things have calmed down can change the tone of the day.  The surgeon not only recognizes the trainee by trying to build on their strength, but the supervising scrub tech also acknowledges the surgeon by providing them positive feedback and recognizes their skills as a teacher.
